# Supplementary material for: A growing bacterial colony in two dimensions as an active nematic
Source: Nat Commun. 2018 Oct 10;9:4190. doi: 10.1038/s41467-018-06370-3 (PMC6180060; doi:10.1038/s41467-018-06370-3)
Supplement: Supplementary file 1 — Supplementary Information [file 41467_2018_6370_MOESM1_ESM.pdf]

A growing bacterial colony in two dimensions as an active nematic
Supplementary Information

D. Dell'Arciprete *et al.*

Supplementary Figures

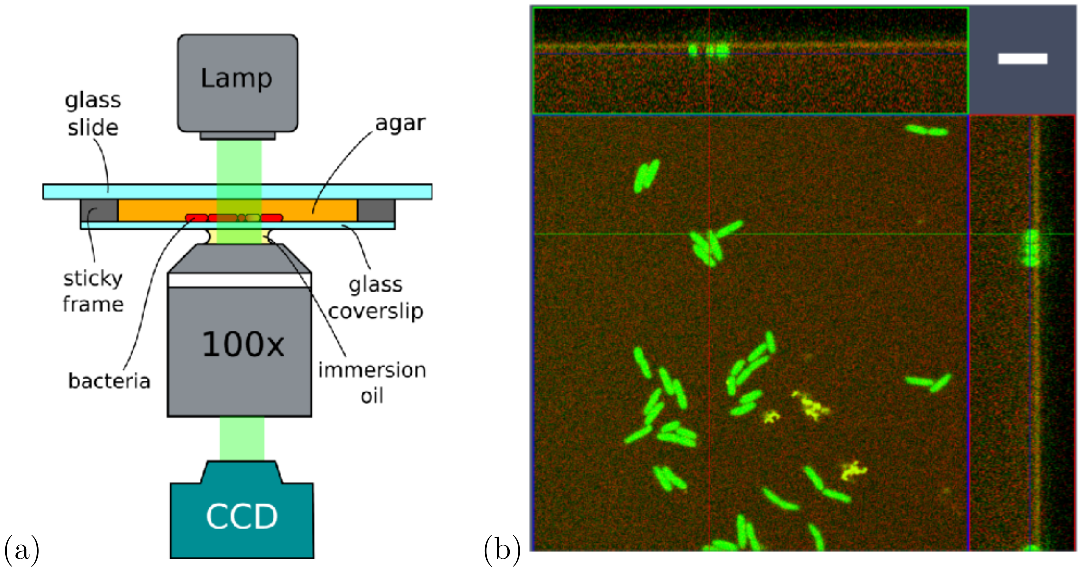

Supplementary Figure 1: **Experimental setup.** (a) Sketch of the apparatus used for time-lapse recording of growing *Escherichia coli* colonies. (b) Confocal image with side projections. Scale bar in top-right corner is 5 µm.

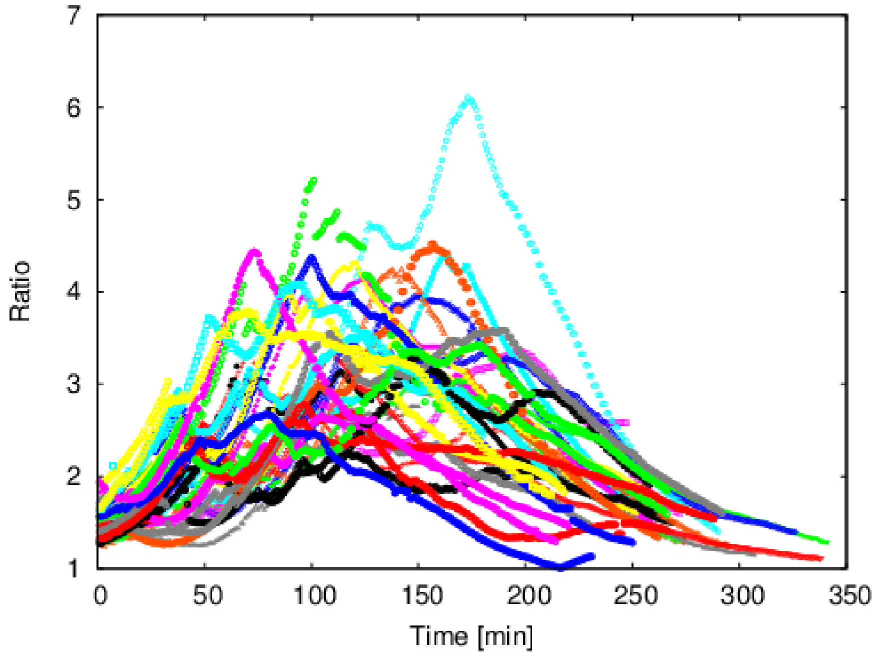

(a)

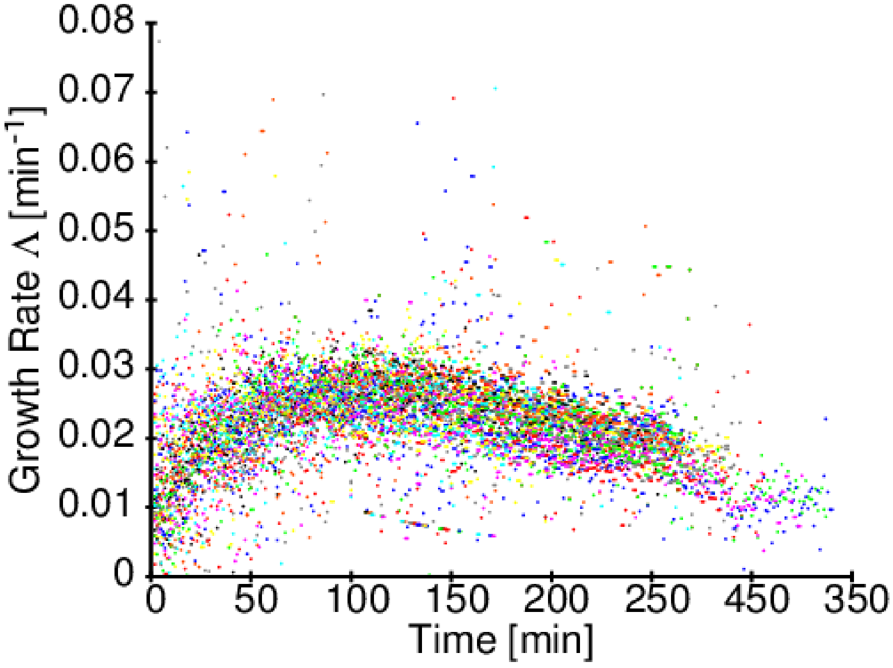

(b)

Supplementary Figure 2: **Shape anisotropy in microcolonies.** (a) Plot of the shape anisotropy as measured by the square-root ratio of the eigenvalues of the gyration tensor over time, for each of the colonies (different colours correspond to different colonies, there is no correspondence between these colours and those used in Fig. 3 of the main text). (b) Plot of the average growth rate, Λ, of 32 individual colonies as a function of time.

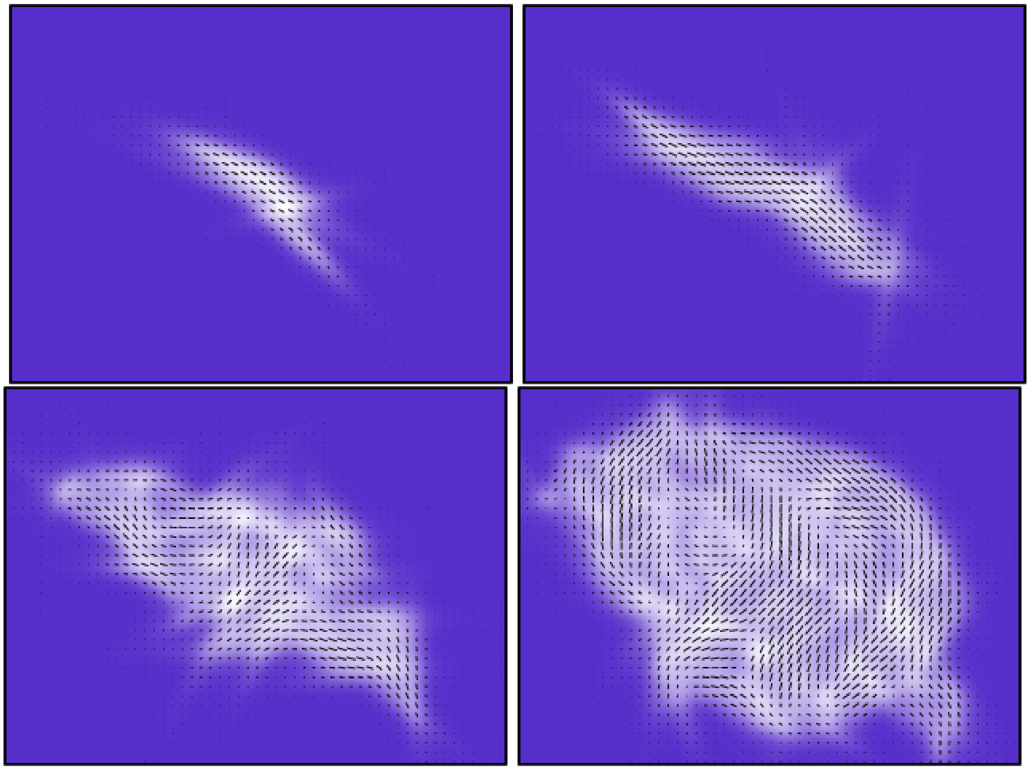

Supplementary Figure 3: **Coarse-grained density and order within microcolonies.** Plots of the density (as a colour plot, blue is least dense), and of the director profile for one of our microcolonies, at different stages of its growth.

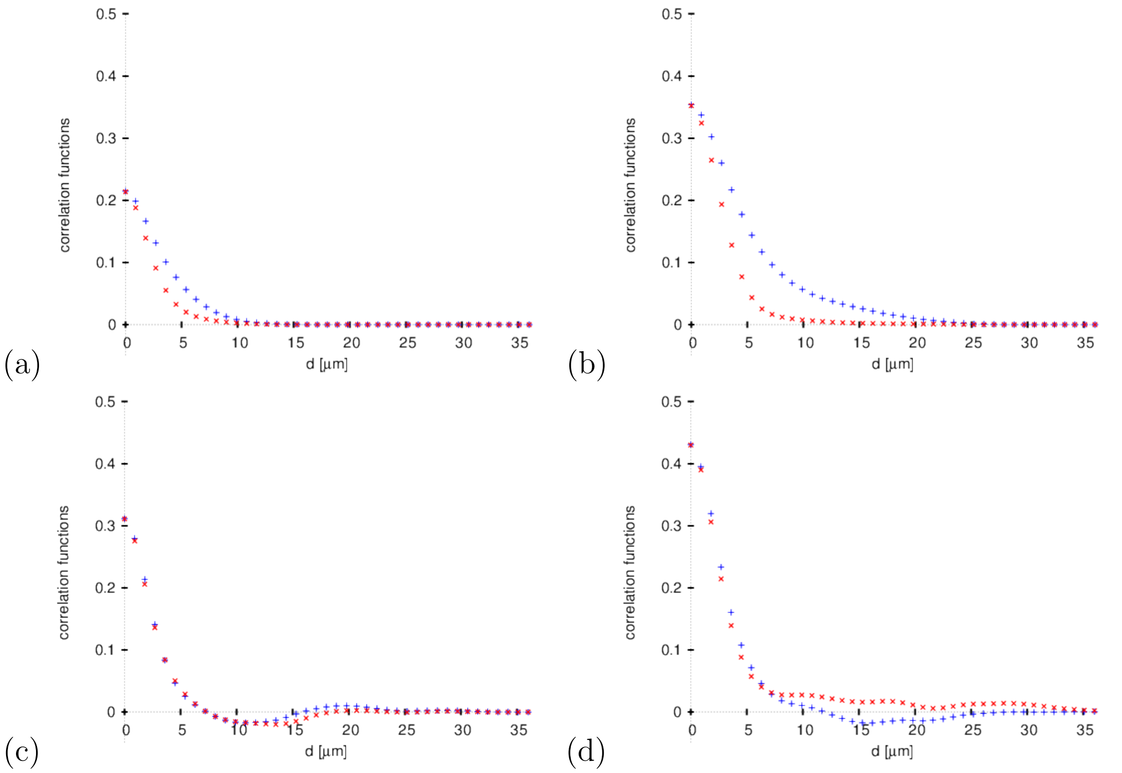

Supplementary Figure 4: **Parallel and perpendicular correlations.** Correlation functions for the colony featured in Figure 1 in the main text, for the four stages shown there, corresponding respectively to (a) $t = 125\,\mathrm{min}$ and $N = 15$ (leftmost, first panel), (b) $t = 171\,\mathrm{min}$ and $N = 56$ (second panel), (c) $t = 216\,\mathrm{min}$ and $N = 164$ (third panel), (d) $t = 261\,\mathrm{min}$ and $N = 475$ (fourth, rightmost panel). $+ = C^{\parallel}(d)$, $\times = C^{\perp}(d)$.

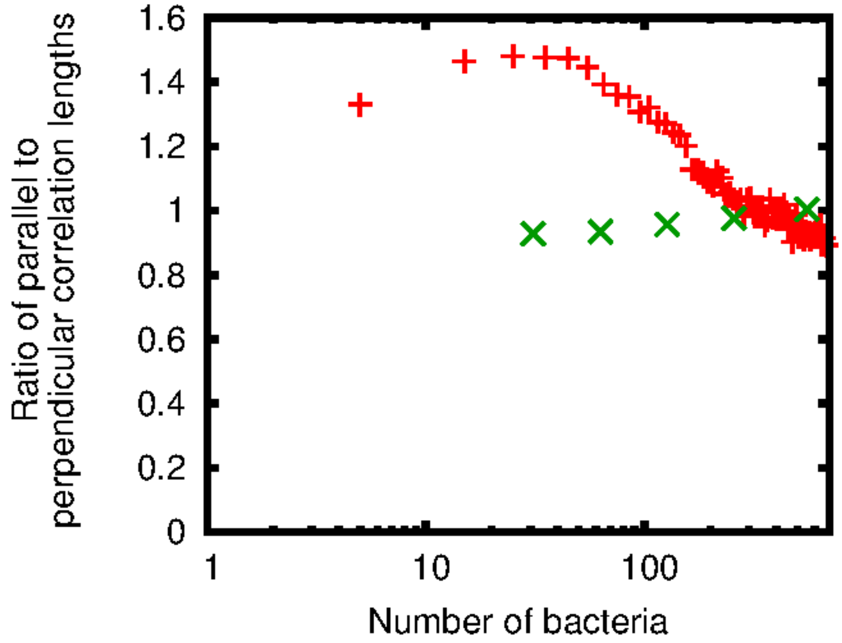

Supplementary Figure 5: **Time evolution of the ratio of perpendicular and parallel correlation lengths.** + = Experimental data, averaged from 32 colonies. × = Data from simulations of passive rods.

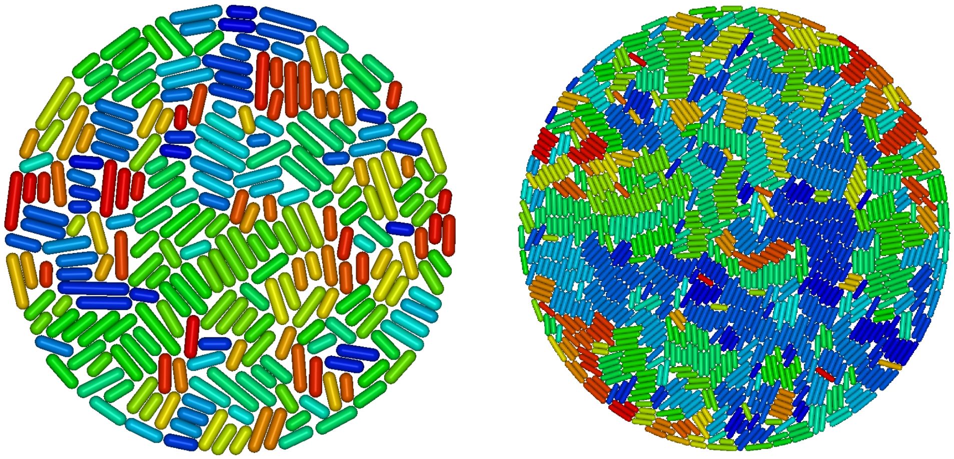

Supplementary Figure 6: **Ordering of passive spherocylinders.** Two examples of a passive spherocylinder "colony", found by Monte-Carlo simulations when subjecting a polydisperse suspensions of spherocylinders to circular compaction.

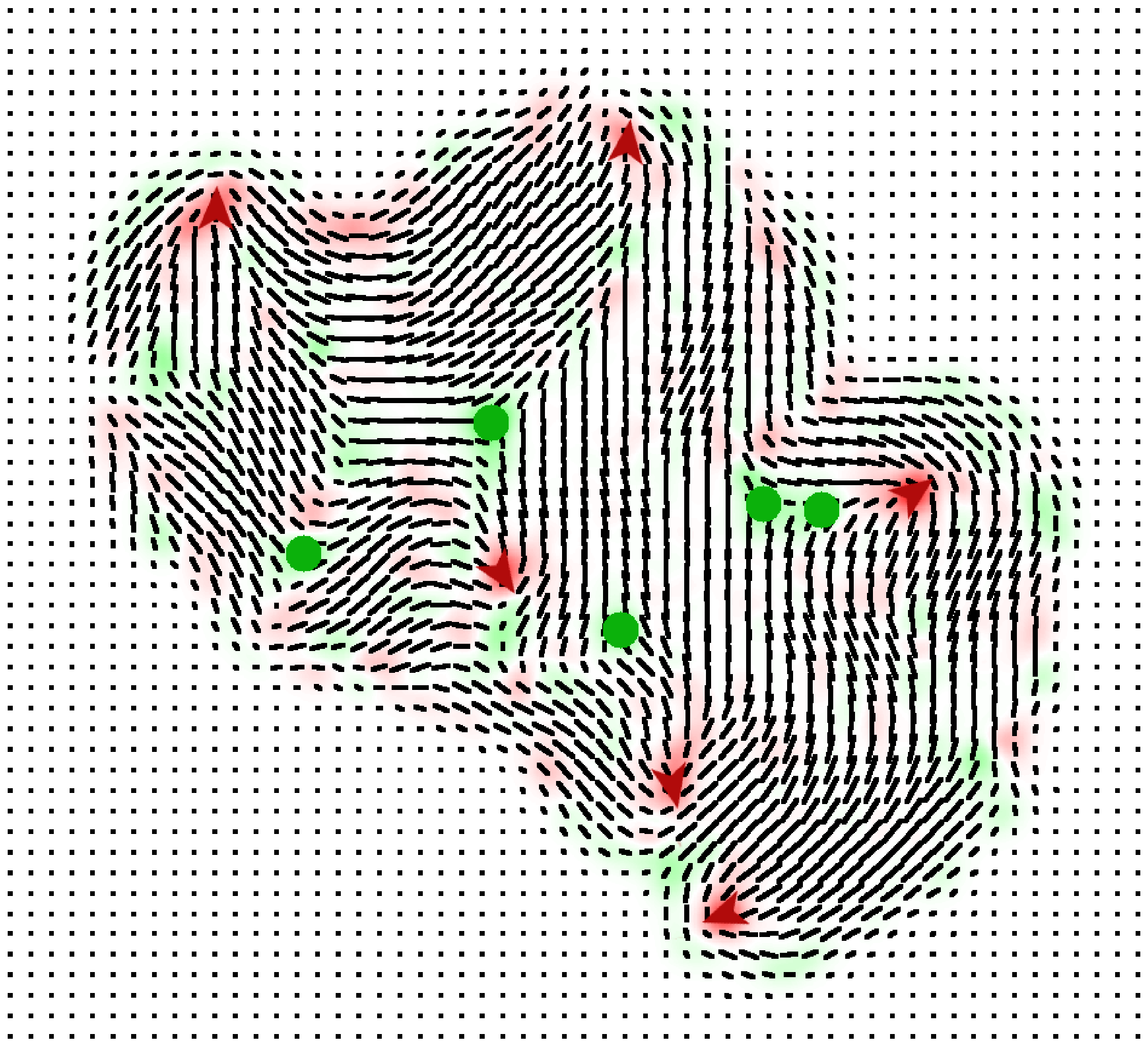

Supplementary Figure 7: **Topological defects.** Map of topological charge density and defects superimposed on a colony at a late stage of growth just before buckling into 3D.

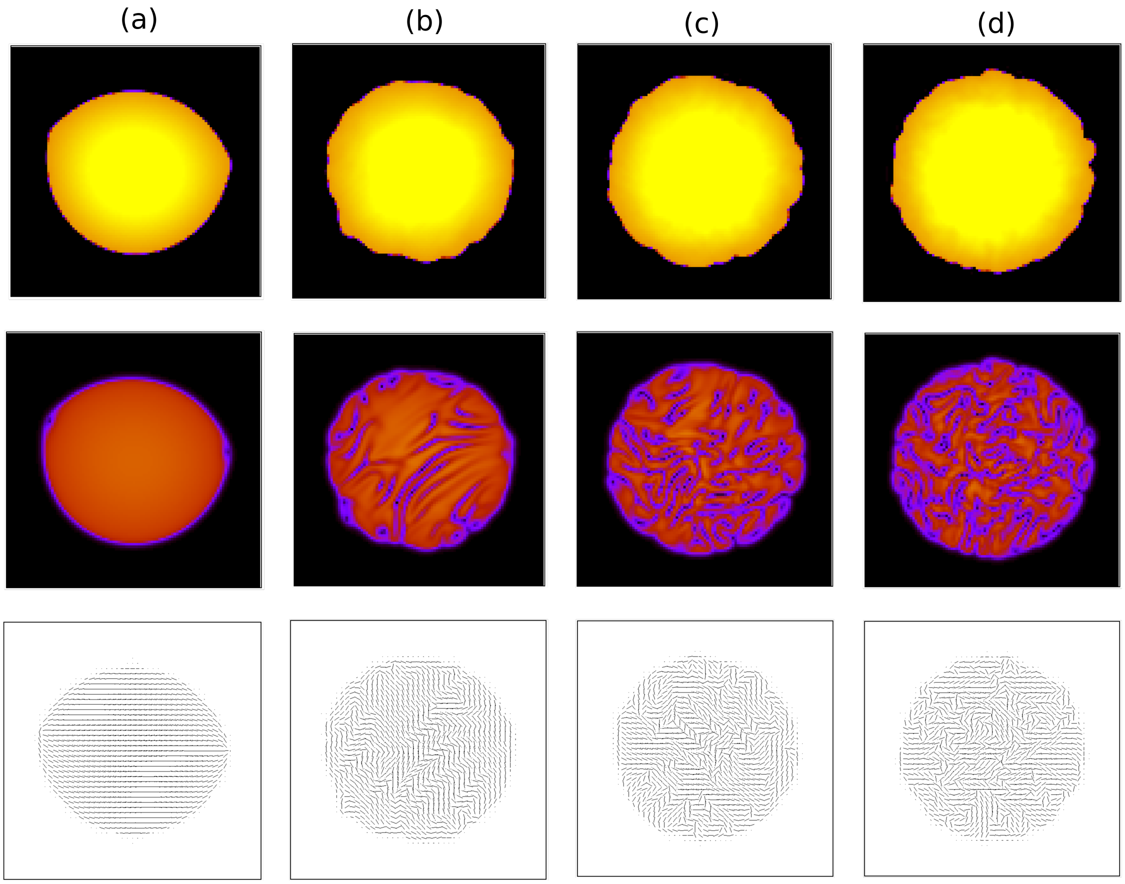

Supplementary Figure 8: **Active field theory simulation snapshots.** Plot of density (top row), scalar order parameter (second row), and director field profile (third row) for a growing bacterial colony after $4,200$ simulation time units, with the same parameters as in Fig. 6 in the main text, except for a_0, which was equal to 0.01 (a), 0.02 (b, as in Fig. 6), 0.025 (c), 0.03 (d). These four values correspond to $\frac{a_0 \rho_0}{\hat{\gamma}\hat{K}}$ equal to 1, 2, 2.5 and 3 respectively. In the contour plots, colours from black to purple to red to yellow denote increasing values of the quantity being analysed.

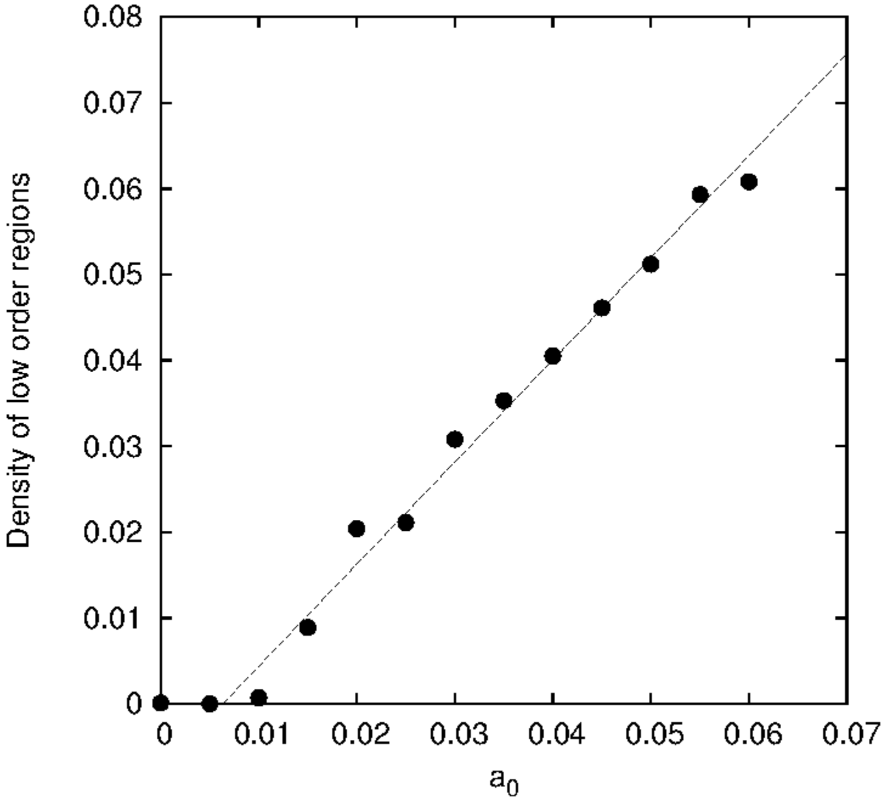

Supplementary Figure 9: **Defect density versus activity.** Plot of the density of points in the simulation domain with local order parameter $S < 0.18 S_{\max}$, where $S_{\max}$ is the maximum over the whole system. The system has grown up to an average overall density of $2\rho_0$, and $L_s = 100$ simulation units.

Supplementary Note 1: Measuring shape anisotropy

In the inset to Figure 1 in the main text, we have shown that the average aspect ratio of the colonies as a function of time is non-monotonic. Here we show data for individual colonies, Supplementary Figure 2(a). While for all colonies the ratio of the major to minor axes shares the non-monotonic average trend, there are significant quantitative colony-to-colony fluctuations.

Not surprisingly, the anisotropy peaks around the time when the colonies show maximal 1D alignment, i.e. when their size is $\sim l_a^2$. After this point, a generic instability sets in and the colony breaks up into aligned domains of size $\sim l_a^2$.

Supplementary Note 2: Measuring space- and time-dependent growth rates

To find the growth rate Λ for a given colony, and at a given time, we measured the number of bacterial cells N and the total projected area A of all bacteria – the latter was approximated by $\sum_i L_i W$ over the cells (with L_i and W the length of the i-th cell and the width, assumed constant over cells). Growth rates were then calculated via the following formula,

$$\Lambda = \frac{1}{A}\frac{dA}{dt}. \tag{1}$$

While the value of Λ is spatially constant to a good approximation within our microcolonies (Fig. 2(a) in the main text), it is clear that it varies over time, Supplementary Figure 2(b). Like other colony characteristics, this statistic has significant colony to colony variations. Interestingly, the growth rate is non-monotonic in time following approximately the same trend as the anisotropy, so that the peak growth rate is observed approximately at the point when the colonies are maximally ordered in 1D. Note that this time variation does not affect any of the conclusions reached in the main text.

Supplementary Note 3: Measuring 'Hubble' cellular flow

To compute the radial velocity (Fig. 2(b) in the main text), we have averaged the velocity of bacteria in all colonies as a function of the distance between the centroid of the colony. More specifically, the calculations require the following steps:

1. Calculate the centroid of the colony based on the unweighted bacterial coordinates, using only trajectories which remain for at least 6 frames.

2. Include only those bacteria within the largest circle centred on this centroid that can be inscribed in the colony.

3. Calculate the displacement between each consecutive frame of each of these bacteria along the radial direction. This gives the radial velocity.

4. Average the radial velocity over the final 10 minutes of each video prior to buckling, binning for distance from the centroid.

Supplementary Note 4: Coarse graining Q-tensor field

As explained in the main text, in order to study the orientational ordering within our colonies, we need to construct a continuum description based on a coarse-grained density field and a coarse-grained Q-tensor field, Eqns. 4 and 5 in the main text respectively. Using these field variables, we can then map out the coarse-grained director profile by identifying the eigenvector corresponding to the larger eigenvalue of the coarse-grained Q-tensor. Supplementary Figure 3 shows the continuum density and director fields of a colony at various stages of its growth.

Supplementary Note 5: Correlation functions of orientational order

In the main text we referred to the use of two-point orientational correlation functions to characterise the short-range order of our colonies. Here we give the precise definitions and plot the results.

We consider the following correlation functions:

$$c^{\parallel}(\mathbf{d}) = \frac{1}{2A} \int d^2\mathbf{r}\rho(\mathbf{r})g(\mathbf{r},\mathbf{r}+\mathbf{d}) \left(\hat{d}_\alpha \hat{d}_\beta Q_{\alpha\beta}(\mathbf{r}) + 1\right), \tag{2}$$

$$c^{\perp}(\mathbf{d}) = \frac{1}{2A} \times \int d^2\mathbf{r}\rho(\mathbf{r})g(\mathbf{r},\mathbf{r}+\mathbf{d}) \left(\hat{d}_\alpha \hat{d}_\beta \epsilon_{\alpha\kappa}\epsilon_{\beta\lambda} Q_{\kappa\lambda}(\mathbf{r}) + 1\right), \tag{3}$$

where $g(\mathbf{r},\mathbf{r}') = Q_{xx}(\mathbf{r})Q_{xx}(\mathbf{r}') + Q_{xy}(\mathbf{r})Q_{xy}(\mathbf{r}')$ and $\hat{\mathbf{d}}$ is the unit vector along $\mathbf{d}$. We then produce radial averages $C^{\parallel}(d)$ and $C^{\perp}(d)$ (binned into annuli of width W), which measure the orientational correlation at a distance d in directions parallel and perpendicular to the director respectively. Supplementary Figure 4 shows $C^{\parallel}(d)$ and $C^{\perp}(d)$ for the colony snapshots in Figure 1 in the main text.

We can see that, between Supplementary Fig. 4(a) and (b), the extent of the correlations grows as the colony elongates. Noting that $W = 0.9\,\mu\text{m}$, we conclude that correlations persist over the scale of several bacteria. Between Supplementary Figures 4(b) and (c), the correlation length shortens as instabilities set in and the colony becomes disordered on a global scale.

Further analysis of these correlation functions and comparison with information obtained from simulations of passive rods (see next section) are facilitated by defining two associated correlation lengths:

$$l_c^{\parallel} = \frac{1}{C^{\parallel}(0)} \int_0^\infty C^{\parallel}(r)dr, \tag{4}$$

$$l_c^{\perp} = \frac{1}{C^{\perp}(0)} \int_0^\infty C^{\perp}(r)dr. \tag{5}$$

We now plot how their ratio varies with time, averaged over 32 colonies, Supplementary Fig. 5 (red pluses), and compare this non-monotonic trend with the behavior of the same ratio from simulations of passive rods (detailed below), Supplementary Fig. 5 (green crosses).

Supplementary Note 6: Monte-Carlo simulations of passive spherocylinders

It is useful to compare and contrast our active growing colonies with corresponding passive (non-growing) collection of spherocylinders, with similar distribution of sizes as within our colonies, and under similar confinement.

To do so, we performed Monte-Carlo (MC) simulations of polydisperse passive spherocylinders. These simulations used a standard Metropolis type acceptance criteria with reduced temperature set to $T^* = 1.0$. To approximate bacterial shape, we used a repulsive spherocylinder model by matching the width D and total length $L + D$, to experimental values. For the repulsion between two particles a Weeks-Chandler-Andersen (WCA) potential [1] was used. Spherocylinders were initialised by setting lengths, centre of mass coordinates and orientations according to experimental readings at a given late stage in colony growth. Coordinates of the centres of mass were then scaled to fit within a circle, with constant radius such that the overall area fraction was approximately 90% (see Supplementary Fig. 6). The system was then relaxed via short simulations of $10^4 - 2.7 \times 10^4$ steps, employing a very soft potential between the particles [2, 3] to eliminate particle overlaps. After the initial relaxation, the system was run for another $200,000$ Monte Carlo sweeps using the WCA potential, leading to configurations such as that shown in Supplementary Fig. 6. Each MC sweep consisted of rotation and translation of each of the particles, a Barker and Watts type algorithm being used for the rotation [4].

Supplementary Figure 6 shows that there is significant ordering perpendicular to the axis of a given spherocylinder, to yield "bookshelf" ordering, as previously observed [5]. There appears, on the other hand, less parallel ordering with respect to the case of the experimental colonies. One way to quantify the differences is to calculate the same orientational correlation functions and associated correlation length as already done for bacterial colonies, and plot the time evolution of the ratio of the two correlation lengths, Supplementary Figure 5. The difference between the active and passive cases is evident.

Supplementary Note 7: Topological charge density and defects

The topological charge density has been defined in the main text, Eqn. 10. Here we show a map of the topological charge density of the colony shown in Figure 1(d) of the main text, Supplementary Fig. 7. Thresholded local maxima and minima in the topological charge density map are identified as $+\frac{1}{2}$ and $-\frac{1}{2}$ defects respectively. These are also shown in the same figure.

Supplementary Note 8: Estimate of frictional drag in bacterial colonies

In the main text, we defined the stress propagation length,

$$l_\sigma = \sqrt{\frac{p}{\hat{\gamma}}\frac{1}{\Lambda}}, \tag{6}$$

where p is a typical pressure scale (which in the main text we argue equals to the yield stress of agarose in our case). To compute l_σ, we need an estimate for $\hat{\gamma} = \gamma L \rho$, where γ is a friction per unit length (units Pa s). To get to this estimate, we use the results in [6], which measured friction forces f of the order of $1-10$nN for bacterial spores on planar surfaces. As spore size is $L \sim 1.5$µm, and the authors moved the spore at $v \sim 2$µms^{-1} (we thank C. Tsouris for communicating this information), we obtain that

$$\gamma \sim f/(Lv) \sim 3 \cdot 10^2 - 10^3 \mathrm{Pa\,s}. \tag{7}$$

Using this value of $\gamma \sim 10^3$ for our *E. coli* bacteria, because $\hat{\gamma} \sim \gamma L \rho$, ρ can be estimated as the inverse bacterial volume $1/V \sim$ µm^{-3}, and $L \sim$ µm, we obtain $\hat{\gamma} \sim 10^{15}$(Pa s)m^{-2}. Clearly, this should be seen as no more than an order-of-magnitude estimate, and it will be important in the future to measure γ more accurately so as to allow more quantitative comparison between theory and experiment.

Supplementary Note 9: Theory for 2D growth-induced alignment

Let us consider the equation of motion for 2D nematodynamics in the absence of any free energy (i.e., no molecular field; see, e.g., [7]),

$$\frac{\partial Q_{\alpha\beta}}{\partial t} + v_\gamma \partial_\gamma Q_{\alpha\beta} = \xi \left[u_{\alpha\beta} - u_{\gamma\gamma}\frac{\delta_{\alpha\beta}}{2} \right] - Q_{\alpha\gamma}\omega_{\gamma\beta} + \omega_{\alpha\gamma}Q_{\gamma\beta}, \tag{8}$$

where $u_{\alpha\beta}$ and $\omega_{\alpha\beta}$ are respectively the symmetric and antisymmetric part of the velocity gradient tensor, $\partial_\beta v_\alpha$. In Supplementary Eqn. 8, the second term on the left-hand side denotes advection, the first and second term on the right-hand side correspond to alignment (ξ is a flow-alignment parameter related to the aspect ratio of the individual particles, here the bacteria, note $\xi \sim S$ in [7]) and rotation due to flow gradients respectively. Note that this equation ensures that $Q_{\alpha\beta}$ remains

traceless and symmetric throughout. If spatial variation of $Q_{\alpha\beta}$ can be disregarded, as we assume here, then we can also neglect the advective contribution on the left-hand side.

Now consider a radially symmetric velocity profile, $v_\alpha = g(r)x_\alpha$, where x_α denotes the Cartesian component of the position vector, and $g(r)$ is a local growth rate (units of inverse seconds). (This is a generalisation of Eqn. 16 in the main text.) Differentiating, we find that the velocity gradient tensor is

$$\partial_\beta v_\alpha = g\delta_{\alpha\beta} + g' r r_\alpha r_\beta, \tag{9}$$

where $r_\alpha = x_\alpha/r$. Now this tensor is symmetric, so that it equals $u_{\alpha\beta}$ (and there is no anti-symmetric part, so that $\omega_{\alpha\beta} = 0$). Upon taking away the trace of Supplementary Eqn. 9 and substituting into Supplementary Eqn. 8, we find

$$\frac{\partial Q_{\alpha\beta}}{\partial t} = \xi g' r \left(r_\alpha r_\beta - \frac{\delta_{\alpha\beta}}{2} \right). \tag{10}$$

We now write $Q_{\alpha\beta}$ as (uniaxial approximation),

$$\begin{aligned} Q_{\alpha\beta} &= 2S \begin{pmatrix} \cos^2\theta - 1/2 & \sin\theta\cos\theta \\ \sin\theta\cos\theta & \sin^2\theta - 1/2 \end{pmatrix} && (11) \\ &= S \begin{pmatrix} \cos(2\theta) & \sin(2\theta) \\ \sin(2\theta) & -\cos(2\theta) \end{pmatrix}, && (12) \end{aligned}$$

where $0 < S \leq 1$ is the magnitude of nematic ordering.

In polar coordinates, (r, ϕ), the right-hand side of Supplementary Eqn. 10 is given by

$$\frac{\xi g' r}{2} \begin{pmatrix} \cos(2\phi) & \sin(2\phi) \\ \sin(2\phi) & -\cos(2\phi) \end{pmatrix}, \tag{13}$$

So that Supplementary Eqn. 10 becomes, in components

$$\begin{aligned} -2S\sin(2\theta)\frac{\partial\theta}{\partial t} &= \frac{\xi g' r}{2}\cos(2\phi) && (14) \\ 2S\cos(2\theta)\frac{\partial\theta}{\partial t} &= \frac{\xi g' r}{2}\sin(2\phi). \end{aligned}$$

We now multiply the first equation by $-\sin(2\theta)$, the second equation by $\cos(2\theta)$, sum them up and rearrange to obtain

$$\begin{aligned} \frac{\partial\theta}{\partial t} &= \frac{\xi g' r}{4S}\left[\sin(2\phi)\cos(2\theta) - \cos(2\phi)\sin(2\theta)\right] && (15) \\ &= \frac{\xi g' r}{4S}\sin\left[2(\phi - \theta)\right]. \end{aligned}$$

Thus, if $g' > 0$, Supplementary Eqn. 15 has a stable equilibrium for $\theta = \phi$ (aster). If $g' < 0$, it has a stable equilibrium for $\theta - \phi = \pi/2$, or $\theta = \phi + \pi/2$ (vortex).

Supplementary References

[1] D. J. Earl, J. Ilnytskyi, and M. R. Wilson. Computer simulations of soft repulsive spherocylinders. *Mol. Phys.*, 99(20):1719–1726, 2001.

[2] Z. E. Hughes, L. M. Stimson, H. Slim, J. S. Lintuvuori, J. M. Ilnytskyi, and M. R. Wilson. An investigation of soft-core potentials for the simulation of mesogenic molecules and molecules composed of rigid and flexible segments. *Comp. Phys. Comm.*, 178(10):724 – 731, 2008.

[3] J. S. Lintuvuori and M. R. Wilson. A new anisotropic soft-core model for the simulation of liquid crystal mesophases. *J. Chem. Phys.*, 128(4), 2008.

[4] M. P. Allen and D. Tildesley. *Computer Simulations of Liquids*. Oxford University Press, 2004.

[5] M. A. Bates and D. Frenkel. Phase behavior of two-dimensional hard rod fluids. *J. Chem. Phys.*, 112:10034–10041, 2000.

[6] H. Kweon, S. Yiacoumi, and C. Tsouris. Friction and adhesion forces of *bacillus thiringensies* spores on planar surfaces in atmoshperic systems. *Langmuir*, 27:14875–14981, 2011.

[7] L Giomi, L Mahadevan, B Chakraborty, and M F Hagan. Banding, excitability and chaos in active nematic suspensions. *Nonlinearity*, 25(8):2245, 2012.
